# A constraint–relaxation–recovery mechanism for stomatal dynamics

**DOI:** 10.1111/pce.13568

**Published:** 2019-05-26

**Authors:** Mareike Jezek, Adrian Hills, Michael R. Blatt, Virgilio L. Lew

**Affiliations:** ^1^ Laboratory of Plant Physiology and Biophysics, Bower Building University of Glasgow Glasgow UK; ^2^ Physiological Laboratory University of Cambridge Cambridge UK

## Abstract

Models of guard cell dynamics, built on the OnGuard platform, have provided quantitative insights into stomatal function, demonstrating substantial predictive power. However, the kinetics of stomatal opening predicted by OnGuard models were threefold to fivefold slower than observed in vivo. No manipulations of parameters within physiological ranges yielded model kinetics substantially closer to these data, thus highlighting a missing component in model construction. One well‐documented process influencing stomata is the constraining effect of the surrounding epidermal cells on guard cell volume and stomatal aperture. Here, we introduce a mechanism to describe this effect in OnGuard2 constructed around solute release and a decline in turgor of the surrounding cells and its subsequent recovery during stomatal opening. The results show that this constraint–relaxation–recovery mechanism in OnGuard2 yields dynamics that are consistent with experimental observations in wild‐type Arabidopsis, and it predicts the altered opening kinetics of *ost2* H^+^‐ATPase and *slac1* Cl^−^ channel mutants. Thus, incorporating solute flux of the surrounding cells implicitly through their constraint on guard cell expansion provides a satisfactory representation of stomatal kinetics, and it predicts a substantial and dynamic role for solute flux across the apoplastic space between the guard cells and surrounding cells in accelerating stomatal kinetics.

## Abstract 2

Models of stomatal function build on the OnGuard platform and our deep knowledge of guard cell transport and metabolism have provided quantitative insights into stomatal function. However, the kinetics of stomatal opening predicted by OnGuard models have consistently failed to match the accelerated opening observed in vivo, thus highlighting a missing component in model construction. Incorporating explicitly a solute flux‐based mechanism of constraint relaxation and recovery between cells surrounding the stoma and the stomatal guard cells accommodates the accelerated stomatal opening kinetics observed in vivo and accurately predicts the slowed kinetics of stomatal opening in guard cell transport mutants.

## 1 INTRODUCTION

Stomata are pores, shaped by paired guard cells on the surface of leaves, that connect the inner air space of the leaf with the surrounding atmosphere. Stomata permit gas exchange, regulating the pore aperture to facilitate CO_2_ entry for photosynthesis while protecting against the drying effects of water loss by transpiration through the pore. Stomata thus play a key role in carbon assimilation for plant growth and in the associated water use efficiency of the plant (Lawson & Blatt, 2014) with substantial influence on crop yields as well as on the global cycles of carbon and water (Berry, Beerling, & Franks, 2010; Franks, Berry, Lombardozzi, & Bonan, 2017; Jasechko et al., 2013).

The factors controlling stomatal movements and their mechanics are the subject of intense study, both because of their intrinsic interest and their immense ecological relevance (Assmann & Jegla, 2016; Hetherington & Woodward, 2003; Jezek & Blatt, 2017; Lawson & Blatt, 2014; Santelia & Lawson, 2016). It is well established that changes in the osmotic load and turgor pressure of the guard cells surrounding each stoma drive the opening and closing of the pore aperture. A network of processes integrate to affect this osmotic load and regulate stomatal dynamics, largely through solute transport and metabolism of the guard cells and their homeostasis (Jezek & Blatt, 2017; Pantin & Blatt, 2018). However, the complexity of this network defies intuitive understanding and has necessitated that its parts are assembled within a comprehensive modelling framework to enable direct comparisons between model predictions and experimental results.

The first comprehensive models of guard cell homeostasis and stomatal dynamics based on the OnGuard platform (Chen et al., 2012; Hills, Chen, Amtmann, Blatt, & Lew, 2012) provided a wealth of novel predictions. Experimental tests (Chen et al., 2012; Wang et al., 2012; Blatt, Wang, Leonhardt, & Hills, 2014; Wang, Hills & Blatt, 2014; Minguet‐Parramona et al., 2016) established the reliability of the representations encoded in the model across a wide range of experimentation and led to a more profound understanding of the complex mechanisms behind many of the responses of guard cells and stomata to environmental change (Wang et al., 2017). Despite these successes, the OnGuard platform has systematically predicted stomatal opening rates slower than that those observed in vivo. Here, we analyse the possible causes of this failing, identify missing OnGuard components that may offer corrective options, incorporate these in the model and experimentally test the predictions of the updated OnGuard platform. Experimental confirmation of the model predictions support a mechanism whereby solute exchanges between the guard cells and their surrounding epidermal cells, with the associated changes in turgor pressure, account for the accelerated stomatal opening kinetics observed in vivo.

## 2 MATERIAL AND METHODS

### 2.1 Growth and gas exchange analysis

Arabidopsis thaliana Col‐0 (wild‐type [wt]), *slac1‐1*, and *ost2‐2* mutant plants were grown, and gas exchange measurements were carried out using LiCOR 6800 gas exchange systems (Lincoln, USA) as described previously (Wang et al., 2012, 2017) with plants preadapted to dark. Plants were grown under 70 mmol m^−2^ s^−1^ light in short‐day conditions (8/16 hr of light/dark) at 22°C/18°C and 55%/70% relative humidity. Seed was harvested at the same time from plants grown together. For gas exchange measurements, all plants were analysed at the same time of the relative diurnal cycle, and measurements were carried out at 400 μl L^−1^ CO_2_. Data were normalized for leaf area using ImageJ v.1.51 (rsbweb.nih.gov/ij/). Unless otherwise noted, all measurements were carried out at 25°C.

### 2.2 OnGuard2 modelling

OnGuard2 was constructed to introduce constraint–relaxation–recovery (CRR) as described above. The construction used the core of the original OnGuard libraries for solute transport, signalling, and homeostasis (Chen et al., 2012; Hills et al., 2012; Wang et al., 2012) with separate assignments of blue and red light (Vialet‐Chabrand et al., 2017).

OnGuard2 models for wild‐type Arabidopsis and the *slac1* and *ost2* mutants were driven through diurnal light:dark cycles as described previously (Blatt et al., 2014; Chen et al., 2012; Wang et al., 2012) and noted in the text, and all model outputs were derived from this cycle. As in the original formulation of OnGuard, light sensitivity was assigned solely to primary, energy‐dependent transport and to sucrose synthesis (Chen et al., 2012; Hills et al., 2012; Wang et al., 2012). To simulate the two mutants in the corresponding models, the transporter component of the SLAC1 current for *slac1* and the [Ca^2+^]_i_ sensitivity of the plasma membrane H^+^‐ATPase for *ost2* were removed (Blatt et al., 2014; Wang et al., 2012). All other parameters were fixed as in the wild‐type model. The outputs of the individual transporters, sucrose and malate metabolism, buffering reactions and transpirational water flux thus responded only to changes in model variables arising from the kinetic features encoded by the model equations and their parameters. A complete list of parameter values used is included in Wang et al. (2017) and Table 2. The OnGuard2 software with CRR option and the models for wild‐type, *slac1*, and *ost2* Arabidopsis are available for free download from www.psrg.org.uk.

### 2.3 Statistics

Results are reported as means ± SE of *n* observations with significance determined by analysis of variance as appropriate, with post hoc analysis (Student–Neumann–Keuls and Tukey), and are indicated at *P* < .05 unless otherwise stated. Note that models built on ordinary differential equations, such as those of OnGuard2, will faithfully reproduce a given set of outputs time and again for any one set of parameters. Statistical analysis of these outputs is therefore meaningless.

## 3 MODELLING RATIONALE AND RESULTS

### 3.1 Role of surrounding cells on stomatal dynamics

**Figure 1:**
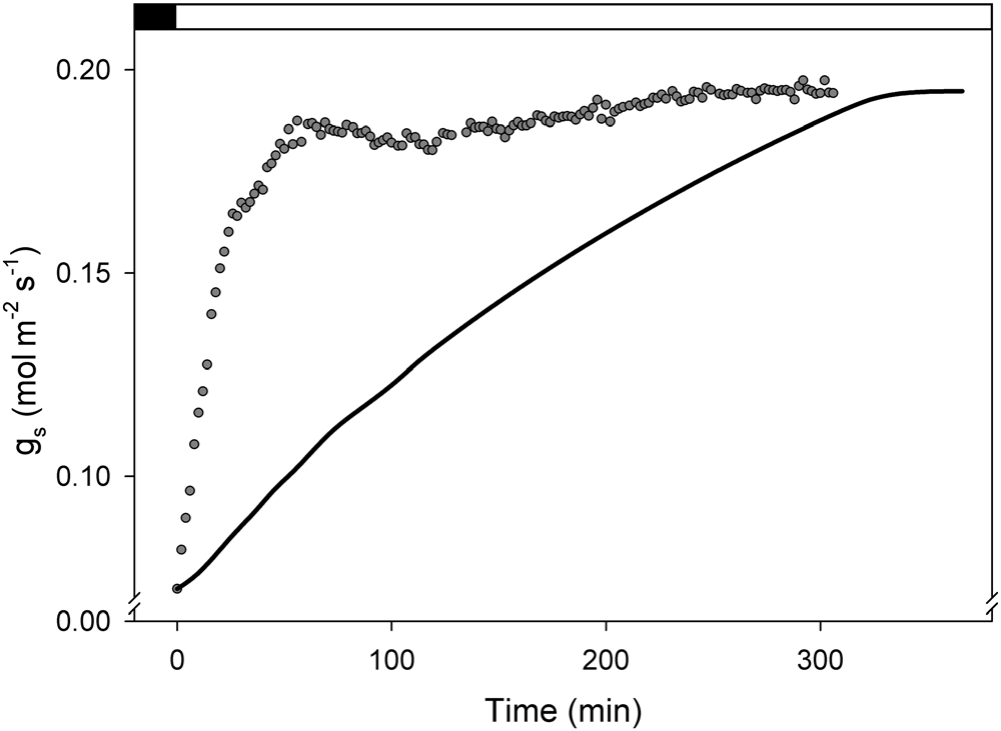
Kinetics of stomatal conductance (g_s_) on transition from dark to light. Data points are from gas exchange measurements of Arabidopsis with a single step at time zero to 400 μmol m^−2^ s^−1^ white light at 400 μmol mol^−1^ CO_2_ and 60 %RH (relative humidity). The solid line is the output of the OnGuard2 simulation for Arabidopsis using the parameters of Wang et al. (2017) and the same environmental conditions with an 8‐hr step in light beginning at time zero. Experimental and simulated half‐times for response are 21 and 132 min, respectively

Rates of stomatal opening observed experimentally vary subject to species and the size of the stomata (Chen et al., 2017; Lawson & Blatt, 2014). In general, however, stomata with kidney‐shaped guard cells, typical of dicotyledonous plants, open in response to light with half‐times on the order of 10–60 min (Iino, Ogawa, & Zeiger, 1985; Lawson, Lefebvre, Baker, Morison, & Raines, 2008; Eisenach, Chen, Grefen, & Blatt, 2012; Wang et al., 2014; Horrer et al., 2016). OnGuard models, for example, for *Vicia* and Arabidopsis, yielded half‐times for opening of 2–4 hr and continue opening through much of the daylight period (Chen et al., 2012; Wang et al., 2012; Wang et al., 2014; Minguet‐Parramona et al., 2016). This discrepancy is exemplified in Figure 1 using experimental data for stomatal conductance, *g*_s_, measured from wild‐type Arabidopsis and the corresponding model output using the wild‐type Arabidopsis parameters from Wang et al. (2017).

Since the pioneering work of Fischer (1968), the net gain of K^+^ ions has been widely recognized to impose a dominant influence on stomatal opening, with malic acid making up between 50% and 90% of the counter ion in different species and inorganic anions, notably Cl^−^, generally making up the rest (Jezek & Blatt, 2017; Willmer & Fricker, 1996). Guard cell K^+^ uptake from apoplastic space is augmented by light‐mediated activation of the H^+^‐ATPase, which promotes a hyperpolarization of the plasma membrane (Jezek & Blatt, 2017). Nonetheless, no physiologically reasonable manipulation of model parameters defining the H^+^‐ATPase, the K^+^ and Cl^−^ (anion) channels, their populations at the plasma membrane, or of other transporters participating in guard cell solute accumulation, could bring the model‐predicted stomatal opening substantially closer to the experimentally observed kinetics. That changes in transporter kinetics within experimentally constrained limits failed to accelerate stomatal opening is noteworthy, because it suggests that controlling factors or signals operating exclusively through guard cell membrane transporters cannot enhance the rate of stomatal opening. The finding is an important model‐derived insight in its own right: it indicates that the missing corrective process of the opening kinetics in the model must be sought among influences external to guard cell transport.

Early work (MacRobbie, 1980; MacRobbie & Lettau, 1980a,b; Bowling, 1987) showed that light‐activated stomatal opening was associated with a shuttle of osmotically active solutes and water to and from epidermal, and in some species subsidiary, cells—hereafter referred to as surrounding cells—to guard cells through the apoplast space, with antiparallel changes in turgor and volume of the surrounding cells. Several other observations echoed these findings as well. Notably, following their quantitative analysis of ion contents, MacRobbie & Lettau (1980) concluded that “in the early stages of opening the changes in potassium are too small to account for the osmotic changes required to open the pore.” On isolating stomata in epidermal peels, in which the surrounding cells were killed, the volume expansion of isolated guard cells was found to occur with substantially lower osmotic solute concentrations than that observed in intact cell preparations with the same apertures (Edwards, Meidner, & Sheriff, 1976; MacRobbie & Lettau, 1980; Cai, Papanatsiou, Blatt, & Chen, 2017). Furthermore, the rate of increase in stomatal aperture was found to decline at higher guard cell turgor pressures (Edwards & Meidner, 1979; Meidner, 1982; Meidner & Bannister, 1979). These findings indicated a limitation on aperture imposed by the constraining properties of the guard cell wall and the mechanical advantage of pressure exerted by the surrounding cells in the epidermis, both points that find support across species (Franks & Farquhar, 2007; Lawson & Blatt, 2014). Thus, it is reasonable to expect that the rate of guard cell expansion and stomatal opening is accelerated by a concurrent, if transient reduction in the constraining pressure exerted by the surrounding cells even if, at higher apertures, this acceleration may be dampened by the constraint of the guard cell wall itself.

**Figure 2:**
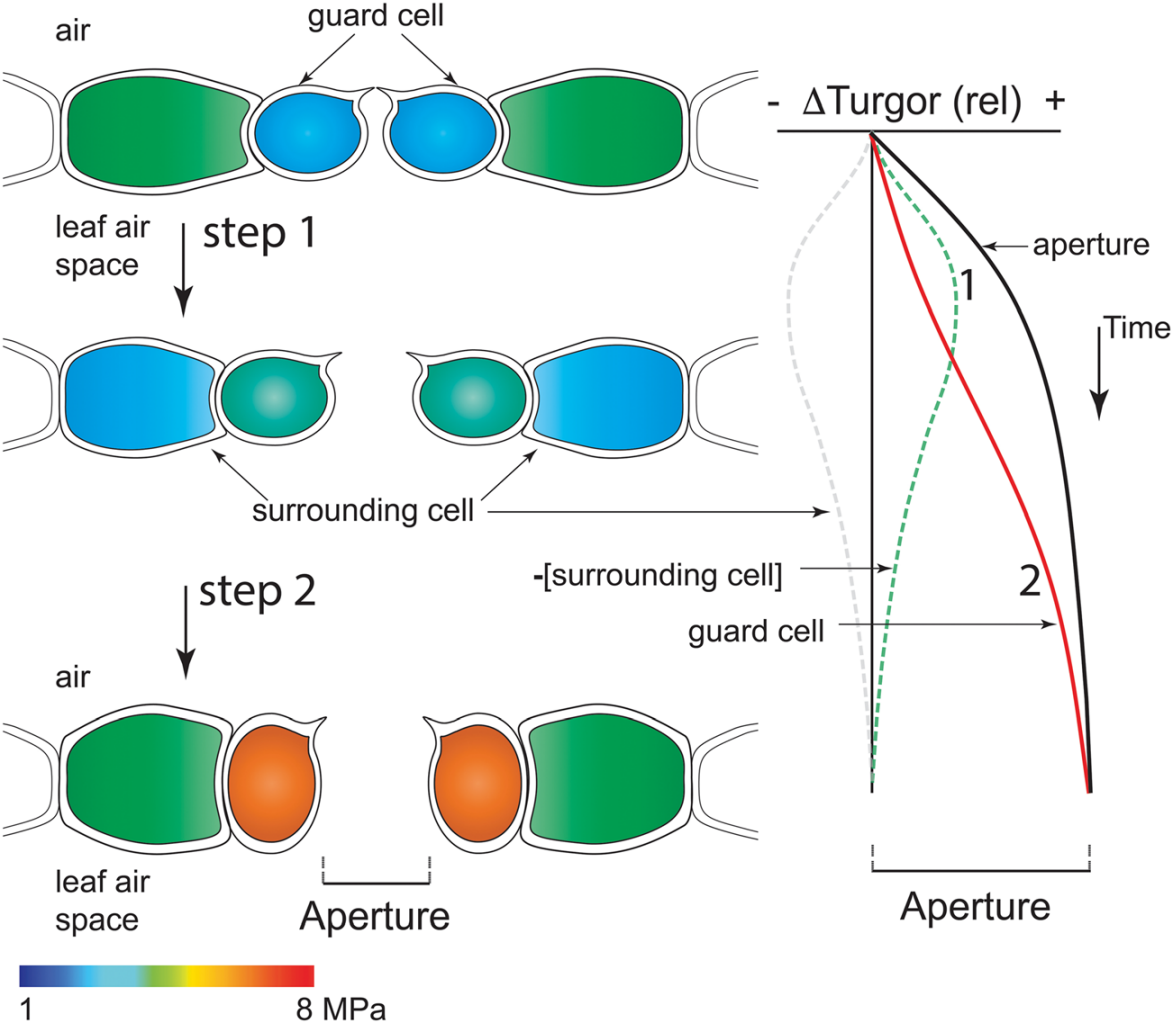
The two‐step sequence proposed for constraint–relaxation–recovery mechanism. Schematic transverse sections through the leaf epidermis (left), showing the guard cells and surrounding cells colour‐coded to indicate the cellular osmotic pressure, presents a rough temporal sequence during opening from closed (top) to fully open (bottom). Scale, 1–8 MPa (below). The corresponding change in aperture (black line) and relative turgor (ΔTurgor) of the guard cell (red line) and surrounding cell (dashed grey line) is plotted in parallel (right). Adding to the aperture kinetics with the guard cell turgor is the inverse of the change in relative turgor of the surrounding cell (‐[surrounding cell] and green dashed line). Note that these two steps contributing to the aperture kinetics start at a common time point

In effect, the antiparallel changes in turgor between the surrounding cells and guard cells may be seen as an initial and partial “volume exchange” between the two cell types, even if a direct exchange does not take place per se but depends on a balance of transport affinities and rates between the two cell types across the buffering matrix of the cell wall. It follows, too, that the surrounding cells may recover via solute and water transport. However, they must do so at rates that are slower than those of the initial changes. Furthermore, although the guard cells continue to accumulate solute, the surrounding cells must recover against the rising turgor of the guard cells. A likely mechanism in the latter case is refilling from surrounding apoplastic solute fed from the transpiration stream (Bowling, 1987; Hedrich et al., 2001; Muhling & Sattelmacher, 1997). Overall, such a process may be seen to comprise a relaxation in constraining pressure on the guard cells followed by a recovery in this baseline constraint, but against the now turgid guard cells and open stoma. These characteristics define an effective CRR mechanism that engages the guard cells, surrounding cells, and their shared apoplastic space in a concurrent, two‐step process of partial exchange in spatial volume and turgor within the epidermal surface (Figure 2).

### 3.2 Assessing constraint-relaxation-recovery with OnGuard

The OnGuard platform utilizes linear, species‐specific relations between guard cell volume, turgor pressure, and stomatal aperture that were originally defined under steady‐state experimental conditions with data for guard cells isolated in epidermal peels (Hills et al., 2012). This phenomenology offered realistic, empirical connections between these variables over a wide range of physiological responses. However, because these relations were derived from measurements of stomata at steady‐state, they do not incorporate any dynamic changes that might be associated with transients in any constraining forces from the surrounding cells.

The basic relationship between turgor pressure (P) and stomatal aperture (A_s_) in the OnGuard platform takes the general form:

(1) $$\text{P}\;=\;mA_{s}+n,$$

where *m* represents the rate of increase in turgor pressure with stomatal aperture and *n* the extrapolated intercept representing the residual turgor pressure on the guard cell at zero stomatal aperture. In all species investigated, the value of *n* was found to be substantially above zero (Edwards & Meidner, 1979; MacRobbie & Lettau, 1980; Willmer & Fricker, 1996). It reflects the baseline turgor pressure of guard cells and the constraining pressure from surrounding cells. The parameter *n* is the obvious starting point for introducing a CRR mechanism. Thus, to introduce time‐dependent dynamics in Equation (1), we consider

(2) $$n=n^{o}\left(1+f\right),$$

where *n* ^*o*^ is the species‐dependent intercept at stomatal closure in the steady state and *f* is the time‐dependent function representing CRR dynamics from the start of opening.

**Figure 3:**
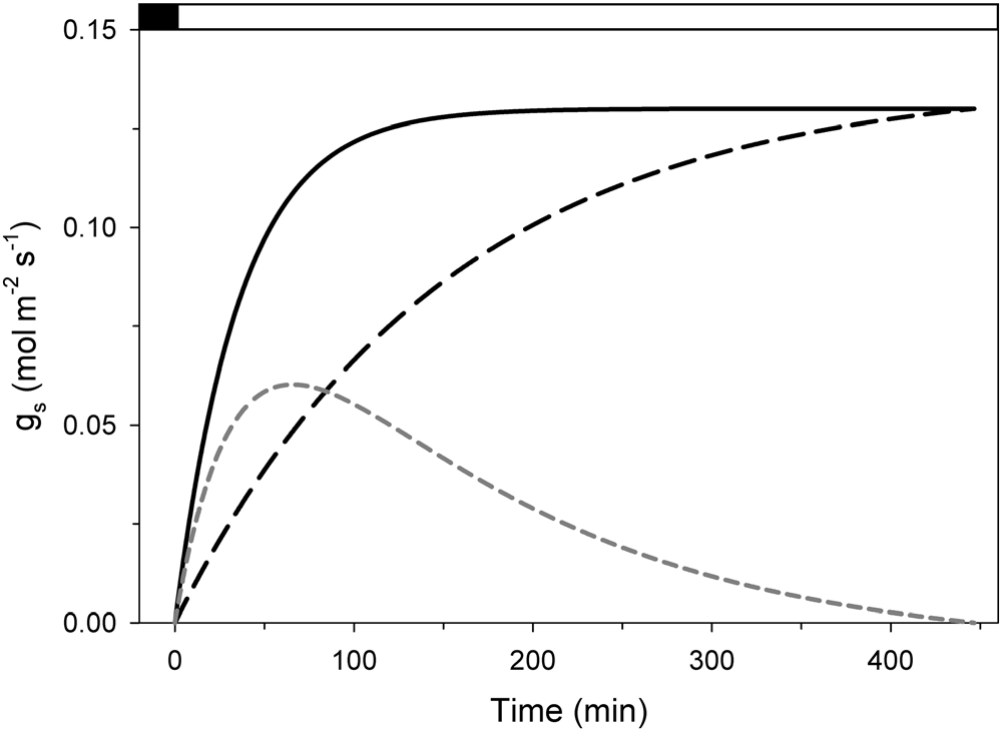
The difference in the relaxation in stomatal conductance (g_s_) between OnGuard2 simulation and experiment describes a biphasic relation. The solid and dashed black lines are single‐exponential fittings to the experimental data and simulation output, respectively, of Figure 1. The dashed grey line is the difference between these two curves. Here, the fittings were adjusted for the offset in initial g_s_

Because *f* is expected to correct the observed discrepancy between measured and predicted stomatal aperture kinetics, a convenient start is to find the temporal characteristic that bridges this gap using the data in Figure 1. This difference will be the mirror image of the basic characteristics sought for *f* in a CRR process such as presented hypothetically in Figure 2. Figure 3 shows the difference between curves fitted to the model and experimental data for the stomatal conductance, *g*_s_ of wild‐type Arabidopsis as shown in Figure 1 with model data calculated using the same wild‐type Arabidopsis model elaborated by Wang et al. (2017) and fitted similarly to a single‐exponential function. The difference here, and in every other example we examined, showed an early maximum and slower decay back to zero as the two curves rejoined later in the daylight period. These characteristics are therefore well approximated by a sum of two exponential curves of the form:

(3) $$f=a\left(1–\left({{e^{-k}}_{2}}^{t}–{{e^{-k}}_{1}}^{t}\right)\right).$$

Here, *a* is a dimensionless term that determines the magnitude of CRR participation in the opening kinetics, and the rate constants *k*_1_ and *k*_2_ may be seen as defining the rates of constraint relaxation and constraint recovery, respectively. In effect, the CRR representation of Equation (3) describes a process whereby the external pressure on the guard cells, *n*, relaxes rapidly and substantially at the start of daylight, reflecting the deflation response of the cells surrounding the guard cells. Surrounding cell turgor and its ensuing effect on guard cell turgor is slowly restored to baseline levels during the day. In other words, the constraint‐recovery rate, *k*_2_, is likely to be as much as one order of magnitude slower than the constraint relaxation rate *k*_1_. The corrective power of *f* on model‐predicted stomatal kinetics when applied to *n* within OnGuard2 is self‐evident from Figures 2 and 3: incorporating *n* with the time‐dependent representation of CRR corrects for the early kinetic deficiencies of the steady‐state assumptions within OnGuard and restores a plateau in stomatal aperture during much of the diurnal cycle, in agreement with common behaviours observed in vivo when no other challenges are imposed.

### 3.3 Identifying components of constraint-relaxation-recovery

Equation (3) identifies CRR empirically with two exponential components. Thus, the immediate question is whether either (or both) of these components are sensitive to transport across the guard cell plasma membrane. To address this question, we compared the rate constants derived for the difference relations from wild‐type plants with those obtained from the *ost2* and *slac1* mutations that are well documented to affect two different ion transporters with very different consequences for ion flux. The *ost2* mutation affects H^+^‐ATPase activity at the guard cell plasma membrane, rendering it insensitive to cytosolic‐free [Ca^2+^] ([Ca^2+^]_i_) (Merlot et al., 2007), whereas the *slac1* mutation eliminates a major pathway for Cl^−^ efflux across the plasma membrane (Negi et al., 2008; Vahisalu et al., 2008).

**Figure 4:**
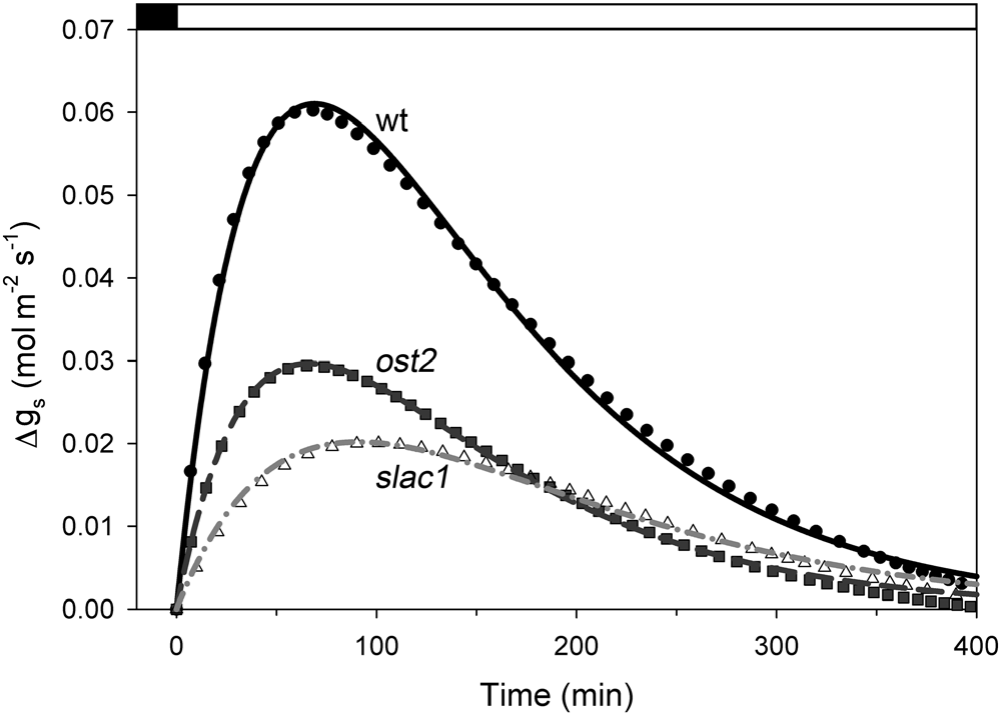
The difference in the relaxation in stomatal conductance (g_s_) between OnGuard2 simulation and experiment for stomata of wild‐type (wt) Arabidopsis (●) and the ost2 (■) and slac1 (△) mutants derived from at least three independent experiments for each line. Here, the data points are the differences between simulation and experimental means and were calculated as in Figure 3. The solid, dashed, and dash‐dotted lines are the results of a joint fitting of these three data sets to Equation (3) with the rate constant k_2_ held in common between all three data sets. Fitted values (in s^−1^): k_2_, 0.0106 ± 0.00006; k_1_, 0.0192 ± 0.0001 (wild‐type), 0.0204 ± 0.0002 (ost2), 0.0114 ± 0.0001 (slac1)

We used the extant models for *ost2* and *slac1* (Blatt et al., 2014;Wang et al., 2012; Wang et al., 2017) and experimental measurements of *g*_s_ to derive the difference relations for each mutant, much as illustrated in Figure 3. Fittings of the mean *g*_s_ over time yielded exponential constants that differed primarily in *k*_1_ between wild‐type, *ost2*, and *slac1* plants. Joint fittings with *k*_2_ held constant between data sets are shown in Figure 4. The results confirm that the differences between experimental and modelled *g*_s_ relations are accommodated by sums of two exponentials with a single slower exponential component that is unaltered between the wild‐type, *ost2*, and *slac1* mutant plants. In other words, although the rate component of constraint relaxation, *k*_1_, is strongly dependent on the transport activity of the guard cells, the rate component of constraint recovery, *k*_2_, is independent of the guard cells. Of course, the empirical rate constants of Equation (3) do not yield further insights into the opening mechanics, but they do inform model construction, as we outline below.

### 3.4 Introducing a mechanistic link for constraint-relaxation-recovery in OnGuard

Given the evidence for an apparent shuttle of solutes between the guard cells and surrounding cells across the apoplast of many species (Bowling, 1987; Edwards et al., 1976; Hedrich et al., 2001; Muhling & Sattelmacher, 1997; Willmer & Fricker, 1996), it is reasonable to expect that constraint relaxation is coupled to the guard cell solute uptake. A review of individual ion fluxes generated over the diurnal cycle (see supplemental material of Chen et al., 2012, for *Vicia* and Wang et al., 2012, for Arabidopsis) showed that model characteristics for net flux for K^+^ across the plasma membrane of guard cells in wild‐type plants singularly rises rapidly to a maximum, consistent with an early draw‐down of solute that might affect the surrounding cells leading to a relaxation in their constraint on the guard cells. We note, too, that model outputs for the *ost2* and *slac1* mutants suggest reduced rates of K^+^ uptake (Wang et al., 2012; Wang et al., 2017), consistent with the values for *k*_1_ derived for each of these mutants.

To introduce a dependence on transport of CRR acting through *n*, we began with a consideration of the ion and water flux through the leaf. The guard cells situate at one end of a diffusional pathway for water and ions that starts at the xylem and passes through the apoplast of the leaf (Buckley, John, Scoffoni, & Sack, 2017; Maiermaercker, 1983; Rockwell, Holbrook, & Stroock, 2014; Wang et al., 2017). This pathway incorporates an apoplastic volume with a limited and static ion buffering capacity, and it communicates primarily with the surrounding cells, only secondarily with the guard cells, which are situated at the very end of the diffusional pathway. Such characteristics are in keeping with our knowledge of the cell wall composition (Bush & McColl, 1987; Grignon & Sentenac, 1991) and the isolation of the surrounding cells and guard cells over the substomatal cavity (Nadeau & Sack, 2002; Papanatsiou, Amtmann, & Blatt, 2017; Renzaglia, Villarreal, Piatkowski, Lucas, & Merced, 2017). It also implies a substantial role for the surrounding cells as a solute reservoir for guard cell transport. In OnGuard, guard cell transport draws on solute within the apoplast; however, the overall osmotic content of the apoplast is assumed to remain constant for any given set of boundary conditions (Chen et al., 2012; Hills et al., 2012). In keeping with this underlying structure, we assume that the fluxes of the relevant solutes do not alter the overall sum of osmotic activity within the apoplast experienced by the guard cells or the surrounding cells. Thus, in line with the long‐established evidence for solute shuttling between surrounding and guard cells (Humble & Raschke, 1971; Raschke & Fellows, 1971; MacRobbie & Lettau, 1980; Bowling, 1987), we posit that the surrounding cells exchange relevant solutes with the apoplast adjoining the guard cells as guard cell transport draws on this pool. Solute loss from the surrounding cells reduces their turgor, which impacts on stomatal aperture through the parameter *n*.

**Figure 5:**
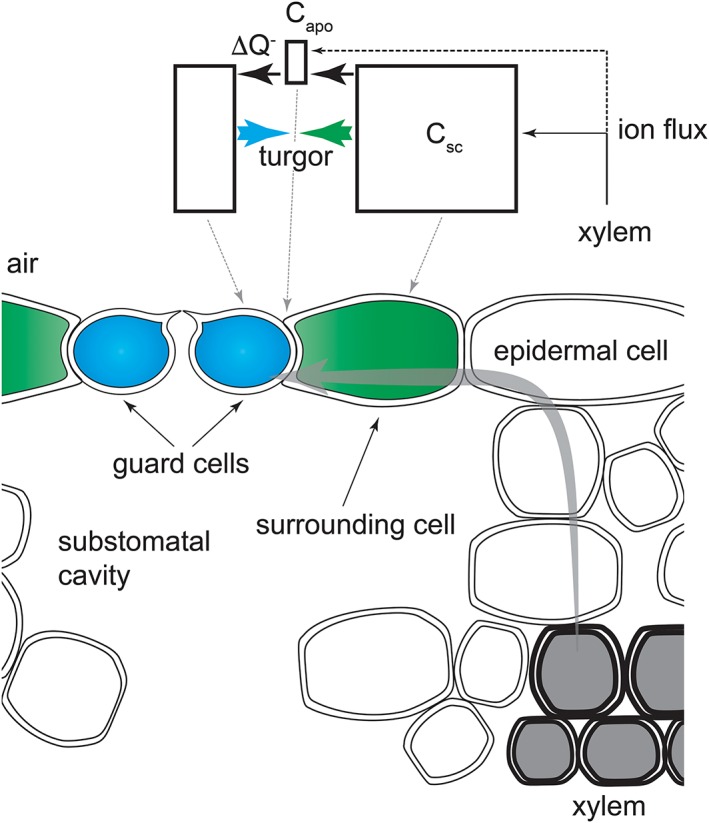
Schematic of solute flow from the xylem to the guard cell. Transverse section through the leaf (below) illustrates the isolation over the substomatal cavity of the guard cells (blue) and surrounding cells (green). Solute and water flow (grey arrow) from the xylem (grey) must pass through the mesophyll and apoplastic space to the epidermis and then along the epidermis to reach the guard cell. The corresponding component compartments (C_apo_, C_sc_) and solute flux of the constraint–relaxation–recovery mechanism are indicated (above) with the opposing turgor of the guard cell and surrounding cell represented by the coloured arrows

A minimum model consistent with these characteristics is included schematically in Figure 5. Here, solute diffuses from tissues beyond the substomatal cavity and maintains the relevant solute content of the compartment, C_sc_, of the surrounding cells. This content is limited by a maximum capacity ^max^C_sc_ that reflects the fully turgid state of the surrounding cells. Solute from tissues beyond the substomatal cavity also adds secondarily to ions in a smaller compartment, C_apo_, which may represent a mobile component of the apoplastic space for the same solutes between the guard cells and surrounding cells. However, we consider C_apo_ to be maintained primarily via transport from the surrounding cells and, like C_sc_, to hold a maximum of relevant solute ^max^C_apo_. Transport by the guard cells is determined as previously described within the OnGuard platform (Chen et al., 2012; Wang et al., 2012; Wang et al., 2017) and draws on C_apo_ and, through this compartment, on C_sc_. In the simplest sense,

(4a) $${C_{\mathrm{apo}}}^{t}={C_{\mathrm{apo}}}^{t-1}+{\mathrm{\Delta Q}}^{-}\left({C_{\mathrm{apo}}}^{t-1}/{}^{\max}C_{\mathrm{apo}}\right)+S_{\mathrm{Fa}}\Delta t$$

and

(4b) $${C_{\mathrm{sc}}}^{t}={C_{\mathrm{sc}}}^{t-1}+{\mathrm{\Delta Q}}^{-}\left(1-{C_{\mathrm{apo}}}^{t-1}/{}^{\max}C_{\mathrm{apo}}\right)+S_{\mathrm{Fc}}\Delta t.$$

Here, C_sc_^*t*^ and C_apo_^*t*^ are the contents of the relevant solutes at time *t*, ^max^C_sc_ and ^max^C_apo_ are the maximum contents of each compartment, and C_sc_^*t*−1^ and C_apo_^*t*−1^ are the contents of the relevant solutes at the preceding time *t* − 1 over the interval Δ*t*; ΔQ^−^ is the net uptake of these solutes by the guard cells over Δ*t* (see also Hills et al., 2012 and Wang et al., 2017); S_Fc_ is the rate of solute uptake by the surrounding cells; and S_Fa_ is the rate of solute feed to C_apo_.

We integrate the fluxes and changes in compartment contents over the series of small time increments that form the core of the OnGuard computational process. By doing so, the relatively simple formulations of Equations (4a) and (4b) yield a pseudo‐exponential decay in the contents of C_apo_ and a corresponding pseudo‐exponential rise to a maximum in the fraction of flux from C_sc_ to C_apo_ that is immediately consumed by flux from the C_apo_ to the guard cell. Thus, just as would be expected of series of first order processes, the kinetics of the net flux into the guard cell draws C_apo_ down to a near‐zero value according to Equation (4a) and the flux into guard cell approaches that of the flux out of C_sc_ according to Equation (4b) until

(4c) $${C_{\mathrm{sc}}}^{t}\approx {C_{\mathrm{sc}}}^{t-1}+{\mathrm{\Delta Q}}^{-}+S_{\mathrm{Fc}}\Delta t.$$

Finally, we scale S_Fa_ to a fraction of S_Fc_ proportional to the respective content maxima ^max^C_sc_ and ^max^C_apo_ so that

(5) $$S_{\mathrm{Fa}}={\left({}^{\max}C_{\mathrm{apo}}/{}^{\max}C_{\mathrm{sc}}\right)}^{.}S_{\mathrm{Fc}}.$$

Thus, the compartment C_apo_ effectively serves as a buffer between the guard cell and C_sc_ as the relevant solutes shuttle between the two cell types.

In principle, solute return from the guard cells on stomatal closure as well as solute drawn from the diffusional pathway will contribute to C_apo_ and refilling of C_sc_. However, both C_sc_ and C_apo_ will depend primarily on diffusion from tissues outlying the substomatal cavity, as C_sc_ and C_apo_ recovery will commonly take place concurrent with solute uptake by the guard cells. This interpretation is consistent with our finding (Figure 4) that the rate of constraint recovery is independent of guard cell transport per se. The rate, S_Fc_, of C_sc_ refilling will be limited by the relevant driving forces, regardless of the immediate source. Thus, in the simplest terms, the rate of uptake must decline with the driving force as C_sc_ approaches the maximum content ^max^C_sc_ so that

(6) $$S_{\mathrm{Fc}}={}^{\max}S_{\mathrm{Fc}}\cdot {\left[\left({}^{\max}C_{\mathrm{sc}}-{C_{\mathrm{sc}}}^{t}\right)/{}^{\max}C_{\mathrm{sc}}\right]}^{b},$$

where ^max^S_Fc_ is the maximum rate of solute uptake by the surrounding cells and, again, ^max^C_sc_ is the maximum content of the fully turgid surrounding cells. Here, the power *b* is included to allow for non‐linearity in the relative rates of refilling; with *b* = 1, the value of S_Fc_ will decline in strict proportion with the fraction of C_sc_ that remains unfilled. It follows that the rate S_Fa_ similarly declines according to Equation (5).

Finally, we adjust *n* with C_sc_^*t*^ relative to the maximum ^max^C_sc_ of the fully turgid surrounding cells to connect constraint relaxation and recovery in *n* to solute flux of the surrounding cells, and we accommodate the non‐linearity of high turgor imposed by the guard cell wall (Edwards & Meidner, 1979; Meidner, 1982; Meidner & Bannister, 1979) by attenuating deviations in *n* introduced the ratio [^max^C_sc_ − C_sc_^*t*^)/^max^C_sc_] as a hyperbolic function of turgor. Thus, we substitute into *f* of Equation (2) so that

(7) $$n=n^{o}\left(1-\tau \left[{}^{\max}C_{\mathrm{sc}}-{C_{\mathrm{sc}}}^{t})/{}^{\max}C_{\mathrm{sc}}\right]\right)$$

and

(8) $$\tau =1/\left(1+{{e^{(P-P}}_{1/2}}^{)\delta }\right),$$

where P_1/2_ and δ are the midpoint and sensitivity coefficient, respectively, for turgor attenuation in constraint relaxation.

Note that the calculations of C_sc_, S_Fc_, τ, and *n* along with C_apo_ and S_Fa_ are incorporated within each time increment of the computational cycle of the OnGuard platform. They represent a mechanistic process of solute transport by the surrounding cells that is coupled to the transport activities of the guard cells through ΔQ^−^ and Equations (4a) and (4b). In keeping with philosophy of the OnGuard platform, the output of OnGuard2 with CRR does not arise from any phenomenological difference in exponentials such as described by Equation (3), but instead, it evolves together with the processes of transport and metabolism that are defined by the sets of equations and their parameters in each OnGuard2 model. Thus, each model becomes a hypothesis under test, to be discarded, validated, or refined by comparisons between model predictions and experimental results.

### 3.5 Validating a link to K^+^ flux for constraint-relaxation-recovery

To assess whether the CRR representation of Equations (2) and (4a–8) can account for the acceleration in stomatal opening, we assigned control of ΔQ^−^ of Equations (4a) and (4b) to the net flux of K^+^ across the guard cell plasma membrane in OnGuard2, combining this variable with a constant ^max^S_Fc_ to calculate *n* at each time interval for Equation (1). We stress that these observations do not rule out an association of ΔQ^−^ with other solutes or their combination. However, as noted above, K^+^ singularly exhibits flux kinetics with an early maximum anticipated for determining the constraint relaxation phase of CRR. Furthermore, K^+^ is the predominant osmoticum that is transported across the guard cell plasma membrane during stomatal movements. Indeed, it proved sufficient to assign control of ΔQ^−^ to the flux of K^+^ for coupling constraint relaxation imposed by the surrounding cells on the guard cells. Assigning control of ΔQ^−^ in constraint relaxation solely to the flux of each of the other major osmotic solutes, to H^+^ and to Ca^2+^, failed to yield a substantial acceleration in stomatal kinetics and transition to a steady‐state.

An obvious prediction of this model is that mutants affected in the capacity K^+^ uptake at the plasma membrane should be affected also in the rate of stomatal opening. In other words, Equations (2) and (4a–8) should accommodate variations in stomatal opening, even if K^+^ uptake is not the primary mutational target. To validate this expectation, we re‐examined the rates of stomatal opening at the start of daylight in wild‐type and in *ost2* and *slac1* mutant Arabidopsis to compare these experimental data with simulations of *ost2* and *slac1* lines using the same parameter sets as described previously for these mutants (Blatt et al., 2014; Wang et al., 2012; Wang et al., 2017) together with the CRR mechanism of Equations (2) and (4a–8). Thus, we asked whether simulations using a single set of CRR parameters and K^+^ flux incorporated in ΔQ^−^ with each of the mutants was sufficient to reproduce the experimentally observed slowing in stomatal opening.

**Figure 6:**
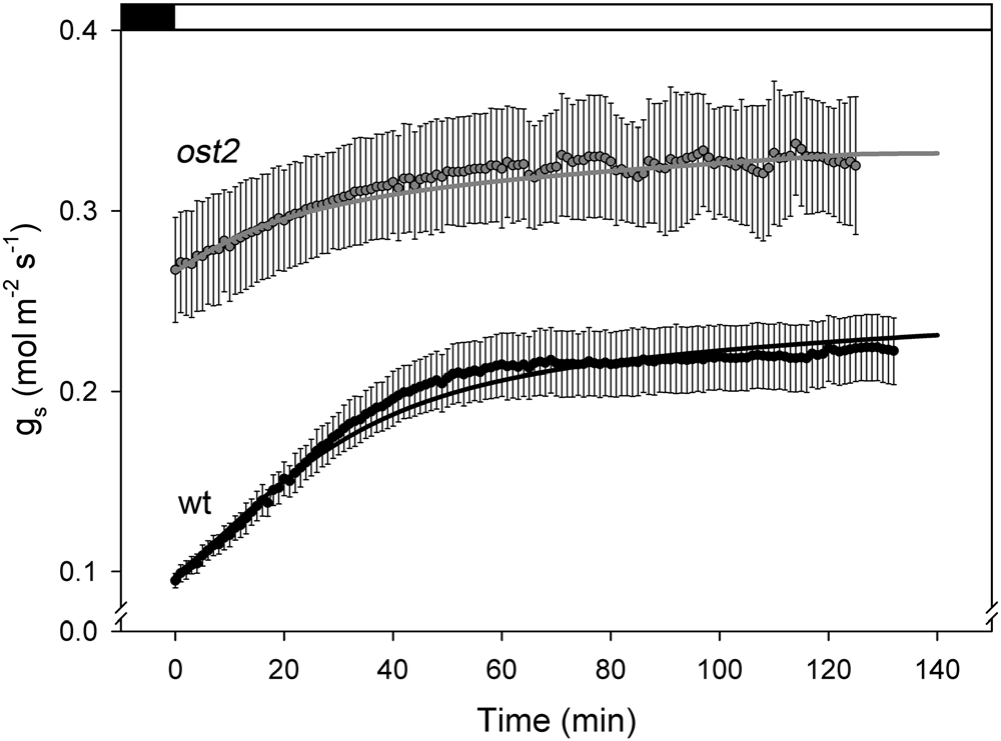
Incorporating constraint–relaxation–recovery (CRR) in OnGuard2 yields stomatal conductance (g_s_) relaxations that recover g_s_ kinetics in both wild‐type (wt) Arabdopsis and the ost2 mutant. Experiment and OnGuard2 simulations were carried out as in Figure 1 with steps from dark to 400 μmol m^−2^ s^−1^ white light at 400 μmol mol^−1^ CO_2_ and 60 %RH (relative humidity). OnGuard2 parameters were those of Wang et al. (2017) and the CRR parameters listing in Table 1. Table 2 provides a comparison of measured and simulated half‐times for g_s_ relaxations from wild‐type Arabidopsis and the ost2 and slac1 mutants

Figure 6 illustrates the kinetics of light‐initiated stomatal opening for the wild‐type and *ost2* mutants with the corresponding OnGuard2 model outputs and incorporating Equations (2) and (4a–8). Table 1 lists the half‐times derived from experimental measurements of *g*_s_ and those obtained from OnGuard2 simulations without and with the CRR correction for the wild‐type Arabidopsis and the *ost2* and *slac1* mutants described before (see Figure 3). Table 2 summarizes the set of parameters that yielded this accelerated stomatal opening kinetics and *g*_s_ in wild‐type Arabidopsis as well as the moderated accelerations evident in both mutants. The results with this single set of parameters showed good fits to the kinetics observed and therefore lend confidence to the connection between CRR and guard cell K^+^ flux. Significantly, the two mutations have a singularly common effect on K^+^ flux, even though the mutations target unrelated transport processes. The *ost2* mutant eliminates the [Ca^2+^]_i_ sensitivity of the H^+^‐ATPase (Merlot et al., 2007) and is modelled by eliminating the ligand dependency of the H^+^‐ATPase to Ca^2+^ (Blatt et al., 2014; Wang et al., 2017); the *slac1* mutant eliminates the dominant plasma membrane Cl^−^ channel (Negi et al., 2008; Vahisalu et al., 2008) and is modelled by setting the SLAC1 component population of Cl^−^ channels to zero (Wang et al., 2012; Wang et al., 2017). Indirectly, both mutations alter the activities of the major K^+^ channels at the plasma membrane, albeit through entirely different mechanisms, and slow stomatal opening (Wang et al., 2012; Wang et al., 2017). Thus, each mutation is modelled through actions that impact on a different transport process, but the consequence of both is a reduction in K^+^ uptake and the rates of stomatal opening (Assmann & Jegla, 2016; Jezek & Blatt, 2017; Wang et al., 2012; Wang et al., 2017).

**Table 1:** The impact of incorporating constraint–relaxation–recovery in OnGuard2 on the half‐times for stomatal conductance (g_s_) increases in the light

	Half‐times (min):		
Arabidopsis	Experiment	OnGuard2	+CRR
Wild‐type	23 ± 1	132	24
*ost2*	29 ± 3	155	26
*slac1*	57 ± 9	254	51

*Note*. Half‐times for experiments taken from exponential fittings to the means ± SE for *g*_s_ of at least three independent data sets with conditions as indicated in Figure 6. Half‐times for OnGuard2 ± CRR taken as the timepoint yielding 50% of the change in simulated *g*_s_ output following the start of the daylight period.

**Table 2:** Common CRR parameters used in OnGuard2 and satisfying the kinetics for opening of wild‐type, ost2, and slac1 mutant Arabidopsis

Parameter	Value	Sensitivity^a^	Defines
^max^C_apo_ (fmol)	1	400	Relevant mobile solute content
^max^C_sc_ (fmol)	10	28	Surrounding cell solute content
^max^S_Fc_ (fmol s^−1^)	0.0035	28	Surrounding cell refill rate
*b*	2	32	Refill relaxation dependence
*n*_*o*_ (atm)	2.5	54	Guard cell turgor at stoma closure
P_1/2_ (atm)	9	8	CRR turgor attenuation midpoint
δ	2	14	CRR turgor sensitivity coefficient

Abbreviation: CRR, constraint–relaxation–recovery.

a Percentage variation yielding a 50% change in the half‐time for stomatal conductance relaxation.

Table 2 also includes the results of a sensitivity analysis of the CRR parameters individually against the kinetics of stomatal conductance with light for the wild‐type and the *ost2* and *slac1* mutant Arabidopsis. These values indicate the variation in each parameter giving a 50% change in the half‐times of relaxation for *g*_s_ in at least one of the simulations for either the wild‐type, the *ost2*, or the *slac1* mutant Arabidopsis. Not surprisingly, the enhanced kinetics of CRR proved most sensitive to variations in parameters defining its attenuation with turgor and to the maximum content and refill rate of the surrounding cells. By contrast, the kinetics proved least sensitive to the content maximum for the secondary (mobile) volume, which affected primarily the delay in onset of CRR.

**Figure 7:**
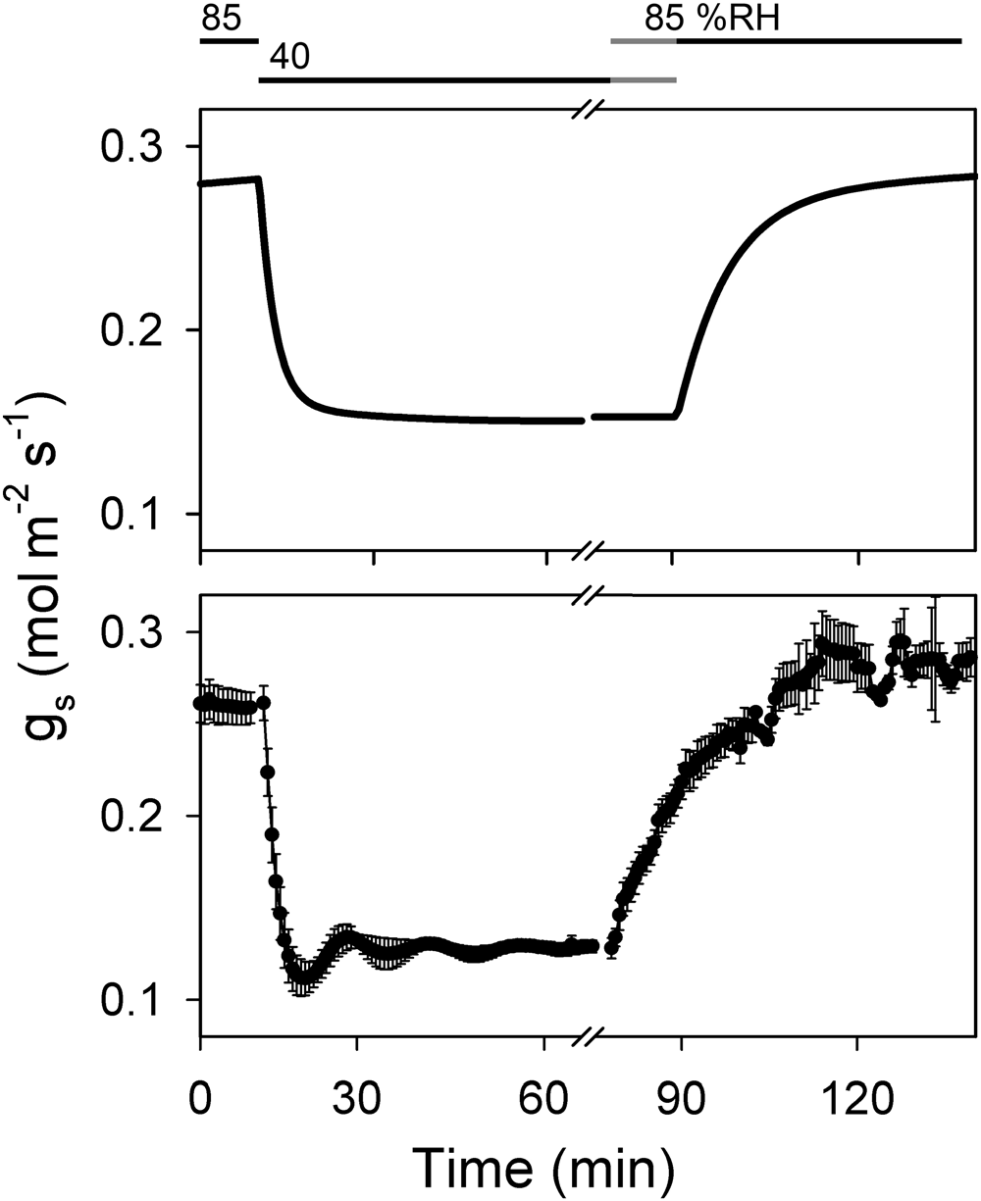
Incorporating constraint–relaxation–recovery in OnGuard2 yields stomatal conductance (g_s_) relaxations that recover g_s_ kinetics measured from wild‐type Arabdopsis with steps in relative humidity (%RH). The OnGuard2 simulation (above) was generated as in Figure 6 incorporating a step between 85 %RH and 40 %RH at 5 hr into the daylight period. The corresponding experimental data (below) are taken from Wang et al. (2017)

Finally, we examined whether the same CRR mechanism is compatible with stomatal kinetics in response to changes in vapour pressure difference (VPD) between the atmosphere and air space within the leaf. By contrast with the effects of light on guard cell membrane transport, in the first instance changes in VPD affect stomatal aperture and conductance directly via rate of transpiration through the stomatal pore and are readily modelled as the consequence of vapour phase equilibration with water in the guard cell wall (Wang et al., 2017). Thus, in OnGuard2, VPD acts initially through the balance of water flux and secondarily on net ion transport. Our expectation was that adding the CRR mechanism in this case should have little or no consequence for model outputs. Indeed, a comparison of experimental data from Wang et al. (2017) and OnGuard2 outputs for Arabidopsis showed that adding the CRR function of Equations (2) and (4a–8) to OnGuard2 had no substantial effect on the dynamics of the output (Figure 7). In short, adding CRR functionality yielded results accommodating stomatal responses across a range of environmental inputs beyond light and are also consistent with previous experimental findings.

## 4 DISCUSSION

We show here that adding a CRR mechanism within the OnGuard platform is sufficient to address the rapid kinetics of light‐induced stomatal opening and to accommodate non‐linear transient processes such as associated with changes in relative humidity. Given the difficulty to explain the opening kinetics on the basis of guard cell solute uptake and turgor changes alone, these results provide strong support for the general participation of a CRR process in stomatal opening. From the viewpoint of a computational treatment, the results assert the role of the parameter *n*, defining the guard cell turgor at zero aperture in the OnGuard platform, as the point of engagement for a realistic CRR representation. In effect, this addition to the platform converts *n* from an invariant constant to a function of time, with relaxation and recovery rates that reflect the changes in constraining pressure that the surrounding cells exert on the guard cells. We demonstrate that this relatively simple description can be coupled to the flux of K^+^ as the predominant inorganic osmoticum transported across the guard cell plasma membrane. The model outputs recapitulate stomatal opening and conductance changes as observed experimentally. Furthermore, they accurately predict the impaired kinetics of stomatal opening with light in the *ost2* and *slac1* mutants known to affect K^+^ flux indirectly through different mechanisms, and they are equally capable of accommodating stomatal aperture and conductance changes with VPD.

### 4.1 The constraint-relaxation-recovery mechanism and fluid dynamics of the stomatal unit

Constraint relaxation and its recovery implies that stomatal dynamics in the intact leaf results from operational units around each stomatal pore that comprise the guard cells with their surrounding cells and the apoplastic space connecting each operational unit with the transpirational path from the xylem to the external atmosphere. Thus, each unit functions as a three compartment system, consistent with the spatio‐anatomical relationships typical of these cells within the leaf epidermis.

In the OnGuard platform, guard cells are treated with all the homeostatic detail afforded by the wealth of quantitative data available for their transport and metabolism (Assmann & Jegla, 2016; Jezek & Blatt, 2017; Santelia & Lawson, 2016). For the surrounding cells, of which we know little that is relevant to any temporal characteristics for transport, the available information allows their representation with comparatively coarse phenomenology. Nonetheless, the simple expedient of coupling transport of the surrounding cells through a small buffering compartment—ostensibly the intervening apoplast—to guard cell transport, with background refilling, clearly accommodates the range of rates in stomatal opening found experimentally. A similar approach with a relatively large reservoir for water flux proved equally effective in predicting unexpected connections between transpiration and guard cell transport and stomatal conductance (Wang et al., 2017). Just as these connections put to rest past arguments around the distinctions between hydropassive and active transport (Pantin & Blatt, 2018), our analysis highlights the plausible coupling in transport between guard cells and the surrounding cells within a defined temporal window.

It is worth noting that a mechanism of inner leaf “drying”—a reduction in the partial pressure of water vapour within the substomatal cavity—as the stoma opens cannot explain the accelerated kinetics observed in vivo. Indeed, the OnGuard2 platform used in this study incorporates explicitly transpirational feedback on the partial pressure of water vapour within the substomatal cavity, its effect on the water potential of the apoplast and, hence, on stomatal aperture (Wang et al., 2017). The consequence is that changes in atmospheric relative humidity leads to rapid changes in aperture and stomatal conductance in the model, just as it does in vivo (see Figure 7). Nonetheless, this connection to water vapour within the substomatal cavity fails to account for the accelerated kinetics of stomatal opening (Figures 1 and 3). Of course, other mechanisms may also accommodate the accelerated stomatal kinetics. For example, there is a substantial body of evidence supporting carbon flow from starch or fructans to sucrose or malate during stomatal opening, and reverse flows during stomatal closure (Dittrich & Raschke, 1977; Outlaw, 2003; Santelia & Lawson, 2016; Santelia & Lunn, 2017), especially under low light (Horrer et al., 2016). This evidence suggests that metabolically generated osmolytes, mainly malate and sucrose, add to stomatal opening kinetics in some circumstances. Contributions from guard cell metabolism and from transport in the surrounding cells are not mutually exclusive, however. Just as we have introduced CRR within the OnGuard platform, combinations with other, temporally governed processes are easily incorporated. The CRR mechanism allows for submaximal accelerations through the parameters defining its coupling to guard cell ion flux. Thus, the challenge becomes one of establishing experimentally the proportional contributions in which such metabolite surges participate in stomatal opening dynamics and under what environmental conditions they apply.

### 4.2 An evolving constraint-relaxation-recovery mechanism within OnGuard

A key feature of the OnGuard platform is its use of temporal increments to calculate changes in guard cell solute transport and metabolism based on the parameter sets defining the underlying properties for each process. Computational modelling of this kind does not predefine a final endpoint. Instead, it integrates the processes within each simulation by incrementing through a series of small steps in time to calculate a progression of steady‐states. In effect, the output of each simulation evolves, determined only by basic physical laws, the equations and their parameters that define the components of the model and, most importantly, the interactions that arise from their functioning over time. It is important to emphasize this distinction between the computational approach, which leads to emergent behaviours, and analytical approaches such as we used initially to identify the temporal shortfall in opening kinetics with earlier OnGuard models (Figures 1, 3, and 4). Although analytical approaches are useful in assessing differences in temporal kinetics “after the fact” and in predicting final steady‐state relationships, they do not inform on how complex processes may develop through the interactions that occur between their components and therefore are limited in their predictive power.

In this respect, it is helpful to consider how transport in the guard cells interacts with the surrounding cells during CRR. For example, at the start of daylight, each time increment promotes the activity of energy‐dependent transport, especially the H^+^‐ATPase at the plasma membrane, according to the model parameters that define its intrinsic coupling to light (Chen et al., 2012; Hills et al., 2012; Wang et al., 2017). Activating the H^+^‐ATPase promotes membrane hyperpolarization and, in turn, an increase in K^+^ influx defined by the voltage dependencies of both the inward‐rectifying K^+^ channel and the K^+^‐H^+^ symport. As guard cell K^+^ influx rises with each time increment, it draws on the available solute of C_apo_ and subsequently of C_sc_ according to Equations (4a) and (4b), reducing *n* and P according to Equations (1) and (7) and thereby also affects stomatal aperture. These changes are understood intuitively from the relations determining K^+^ flux and the empirical relationship between *n* and guard cell turgor.

Increasing guard cell volume through *n* and P has further effects, however, as the reduced guard cell turgor also increases guard cell volume according to the Van't Hoff relation (Hills et al., 2012), which leads to a proportional dilution of all solutes in the guard cell. These include [K^+^] in the cytosol, which increases the driving force for K^+^ influx as well as further reducing *n* and P through Equations (1), (2), and (4a–7). The volume increase also suppresses [Ca^2+^]_i_, which in turn will have multiple actions through the equations and their parameters defining the fluxes of all [Ca^2+^]_i_‐sensitive transporters, the H^+^‐ATPase, inward‐rectifying K^+^ channels, Cl^−^ and Mal channels, among others. Each of these transporters will affect the corresponding ion fluxes and, consequently, the total osmotic solute content of the guard cell, on balance further enhancing solute uptake. Thus, the incremental changes introduced through relaxation of the constraint of the surrounding cells accelerates stomatal opening, and hence *g*_s_, in a manner that evolves substantially beyond what might be expected of the relaxation defined by a consideration of Equations (1) and (4a–8) alone.

Finally, it is important to note that the CRR process, as implemented in the OnGuard platform, is concurrent with guard cell membrane transport and solute uptake, even if the consequence is to give the appearance of a two‐step sequence of events. A simple assessment of the changes in turgor and aperture might otherwise suggest that the early stage of stomatal opening is reliant on constraint relaxation and the later stage is reliant on solute accumulation in the guard cells (Figure 2). This is not the case and the observations derived from the OnGuard platform here are significant: we demonstrate that the two events are accommodated through a single, integrated process in which both events arise concurrently. In other words, constraint relaxation and constraint recovery do not require triggering independently. It would otherwise be tempting to interpret sequential events, such as those of turgor changes between cells with relative humidity (Mott, 2007; Shope, Peak, & Mott, 2008), without considering the temporal kinetics of each.

## AUTHOR CONTRIBUTIONS

A. H. developed the OnGuard platform with M. R. B. and V. L. L.; V. L. L. initiated the constraint–relaxation–recovery studies and developed the computational approach with A. H. and M. R. B.; M. J. carried out experiments and refined the models with M. R. B.; all authors contributed to writing the manuscript.
